# Emergency medicine: navigating the challenges of complex patients

**DOI:** 10.3325/cmj.2024.65.467

**Published:** 2024-10

**Authors:** Adis Keranović, Višnja Nesek Adam, Anđela Simić, Dorja Vočanec, Aleksandar Džakula

**Affiliations:** 1 Zagreb University Hospital Center, Zagreb, Croatia; 2 Sveti Duh University Hospital, Zagreb, Croatia; 3 Teaching Institute for Emergency Medicine of Varaždin County, Varaždin, Croatia; 4 Center for Health Systems, Policies and Diplomacy, Andrija Štampar School of Public Health, University of Zagreb School of Medicine, Zagreb, Croatia; dvocanec@snz.hr

## The changing nature of emergency medicine

Emergency medicine specialists often say that their specialty consists of “the best and most interesting 15 minutes of every other specialty.” Indeed, emergency situations exist in almost every field of medicine, requiring an optimal application of knowledge, often in extreme situations characterized by limited time and resources (1). It is widely believed that emergency medicine deals only with the most urgent and dramatic cases that need to be resolved in the shortest possible time frame. However, a simple glance at the waiting rooms of hospital emergency medical departments shows that reality is far from this dogmatic view. Even with the sound of ambulances bringing the most urgent patients to hospitals, the impression left by the waiting rooms of emergency medical departments is far from the definition of “urgent,” “specific,” and “resolvable in a short amount of time” (2).

Emergency medicine, which originally developed around its primary function of providing urgent care to patients with life-threatening conditions, has increasingly started dealing with patients suffering from a wide array of symptoms or concerns. This is not unusual or unexpected as it is part of the broader changes happening throughout the health care system. However, in emergency medicine, these shifts are more visible and place a greater strain on daily operations (3). They are largely a consequence of demographic changes, including the increasing proportion of older adults, people living alone, and individuals struggling with daily activities. At the same time, patients are undergoing increasingly complex medical treatments that extend life and improve quality of life through the use of modern technology, both at home and remotely. A notably high proportion of these complex patients visits emergency medical services (4). The profiles of these patients often include not only comorbidities, frailty, and other medical conditions but also social, psychological, and intellectual factors (5).

Therefore, emergency medicine no longer provides only solutions for acute conditions in a short time frame – it now faces a much broader range of challenges, including those related to demographic shifts and the complex health care needs of today’s society. Additionally, emergency medical services lack adequate gatekeeping mechanisms to regulate the inflow of patients and reduce unnecessary visits. In fact, the opposite is happening. As access to health care becomes increasingly limited and waiting lists grow throughout the health system, emergency medical services are often seen as the only available, if not the easiest, shortcut to health care.

## “Hallway medicine” and other symptoms of systemic pressure

In response to these trends, the term “hallway medicine” has emerged, highlighting the widespread practice of treating patients in inappropriate conditions, often in hallways or other improvised spaces. The practice occurs due to the increasing pressure on hospital emergency medical services, the complexity of patients' medical and social conditions, and unclear protocols regarding hospital admissions, discharges, or the continuation of care in other institutions. “Hallway medicine” occurs in the space between hospital emergency departments and inpatient wards, with care for these patients most often provided by emergency medicine specialists. This type of patient management brings with it a range of risks: professional, organizational, legal, and ethical, placing both patients and health care workers in a difficult position (6,7).

Addressing the challenges of emergency medicine today requires a fundamental shift in system organization. Emergency medical services are burdened by a growing number of complex patients, who often present with interconnected health and social issues. This rise in complex cases cannot be effectively managed within the current organizational models, leading to staff overload, burnout, and a declining interest in emergency medicine as a specialty. The inadequate allocation of resources and the unclear distinction between urgent and non-urgent cases further worsen the situation. All this creates an extremely challenging work environment for doctors and nurses in emergency medical services. In addition to the physical and emotional burnout of health care workers, the appeal of the profession itself is becoming a concern. Emergency medicine, once attractive to young doctors, is now perceived as a highly stressful and thus less appealing career choice. This leads to staffing shortages, creating a vicious cycle – fewer staff increases the workload for those who remain, which further discourages potential recruits.

## Moving from reactive to proactive and preventive measures

Addressing challenges in emergency medical services by strengthening other health care sectors, such as primary care, public services, helplines, and case management, has often been proven useful globally, but it has primarily been a reactive approach. Most of these initiatives have focused on “harm reduction,” ie, mitigating the consequences rather than solving the root causes. However, the scale and complexity of the challenges faced by emergency medical services today demand a more comprehensive approach. Interventions must be designed to prevent problems from reaching emergency medical departments in the first place. This means that emergency medical services must be positioned within a broader framework of health care. Only then can emergency medical services once again become a place where urgent situations are handled effectively and efficiently, which would relieve the strain on resources and staff, and ensure a sustainable system (8).

Professionals and managers in emergency medical services today need to broaden their focus and think on a wider scale. Their work should not be limited to emergency medical interventions but should extend to prevention and collaboration across all levels of the health care system, including patients, their caregivers, and other health care workers. This approach involves reducing the risk of emergency medical situations, promoting preventive interventions, empowering patients with self-care strategies, and utilizing telemedicine solutions for remote assistance. Technological and organizational tools must enable interventions in a way that minimizes the burden on both the system and health care workers, particularly when dealing with complex patients.

The creation of large hospital emergency medical departments and the modernization of outpatient emergency medical services was once a step forward. Yet, this was only the initial phase. These advancements have actually exposed much deeper and more complex problems that emergency medicine now faces. Reactive and partial solutions are no longer enough. A comprehensive and proactive approach is needed, one that addresses the entire health care system with the goal of easing the pressure on emergency medical services and improving the overall quality of care. Thus, a shift from reactive to proactive and preventive measures has become essential in ensuring the sustainability of emergency medical care.
